# Acceptability of Financial Incentives for Health Behaviours: A Discrete Choice Experiment

**DOI:** 10.1371/journal.pone.0157403

**Published:** 2016-06-17

**Authors:** Emma L. Giles, Frauke Becker, Laura Ternent, Falko F. Sniehotta, Elaine McColl, Jean Adams

**Affiliations:** Institute of Health & Society, Newcastle University, Newcastle upon Tyne, United Kingdom; University of Pennsylvania, UNITED STATES

## Abstract

**Background:**

Healthy behaviours are important determinants of health and disease, but many people find it difficult to perform these behaviours. Systematic reviews support the use of personal financial incentives to encourage healthy behaviours. There is concern that financial incentives may be unacceptable to the public, those delivering services and policymakers, but this has been poorly studied. Without widespread acceptability, financial incentives are unlikely to be widely implemented. We sought to answer two questions: what are the relative preferences of UK adults for attributes of financial incentives for healthy behaviours? Do preferences vary according to the respondents’ socio-demographic characteristics?

**Methods:**

We conducted an online discrete choice experiment. Participants were adult members of a market research panel living in the UK selected using quota sampling. Preferences were examined for financial incentives for: smoking cessation, regular physical activity, attendance for vaccination, and attendance for screening. Attributes of interest (and their levels) were: type of incentive (none, cash, shopping vouchers or lottery tickets); value of incentive (a continuous variable); schedule of incentive (same value each week, or value increases as behaviour change is sustained); other information provided (none, written information, face-to-face discussion, or both); and recipients (all eligible individuals, people living in low-income households, or pregnant women).

**Results:**

Cash or shopping voucher incentives were preferred as much as, or more than, no incentive in all cases. Lower value incentives and those offered to all eligible individuals were preferred. Preferences for additional information provided alongside incentives varied between behaviours. Younger participants and men were more likely to prefer incentives. There were no clear differences in preference according to educational attainment.

**Conclusions:**

Cash or shopping voucher-type financial incentives for healthy behaviours are not necessarily less acceptable than no incentives to UK adults.

## Introduction

Healthy behaviours such as not smoking, regular physical activity, and taking part in vaccination and disease screening are important determinants of health, morbidity and mortality.[1, 2] However, engagement in these behaviours remains far from optimal. Worldwide, physical inactivity and dietary risk factors account for around 10%, and tobacco 6%, of disability adjusted life-years lost.[2] In the UK, comparable figures are 14% and 12% respectively.[3] Only 51% of UK adults in at-risk groups receive influenza vaccinations;[4] and 20–30% do not engage in cancer screening.[5] In response to these findings, national and international public health strategies include maximising healthy behaviours as core components.[6–8]

One method of encouraging healthier behaviours, that has received significant recent attention, is personal financial incentives.[9–11] These have been defined as financial rewards provided contingent on behaviour change.[10, 12, 13] Financial incentive interventions for healthy behaviours (termed ‘financial incentives’ hereafter) are increasingly used, encouraged, or being considered, by governments around the world. Large programmes exist in some low and middle income countries incentivising a range of maternal and child health behaviours.[14] The Affordable Care Act in the USA allows insurers to offer contingent incentives up to a value of 30% (50% if targeting tobacco) of the cost of insurance plans.[15] On-line, websites such as www.stickk.com allow users to incentivise themselves to achieve almost anything, including their health behaviour goals.

A number of systematic, and other, reviews support the use of financial incentives.[10, 16–23] Non-systematic reviews have reported that financial incentives are more effective for ‘one off’ behaviours such as attending for screening and vaccination, than more complex behaviours such as smoking cessation.[17, 19] However, this is not confirmed in systematic reviews. Systematic reviews find that the effects of financial incentives do not vary according to incentive value or target behaviour, but may be larger in more deprived groups.[10, 16] Whilst these systematic reviews find prolonged effects of continuing incentives, effects after intervention removal appear to decrease over time.[10, 16, 23]

Despite this positive evidence of effect, there are concerns that financial incentives remain unacceptable to the public, potential recipients, those involved in front-line health promotion delivery, and policymakers.[9, 11, 24] Without widespread acceptability, financial incentives are unlikely to be widely implemented[25]–meaning their potential will not be achieved. Key concerns with financial incentives identified in qualitative research include a perception that they reward ‘bad’ behaviour, are socially divisive and ineffective, and that they are too easy for participants to manipulate or ‘game’.[11, 26–28]

Whilst there is much concern about the acceptability of financial incentives, there is less primary evidence describing this. One systematic review of both empirical studies and scholarly writing found substantial scholarly concerns about the ethics and practicalities of financial incentives.[11] A number of surveys of the public were also included, but the review identified little in-depth exploration of what aspects of financial incentives for healthy behaviours are, and are not, acceptable. Greater understanding of what influences the acceptability of financial incentives may help in designing interventions which are both acceptable and effective.

Qualitative research has identified a range of concerns that stakeholders have about financial incentives,[26, 27] but cannot determine the relative importance of these. Discrete choice experiments (DCEs) are a quantitative method for exploring stated, rather than revealed, preferences for the characteristics of services, interventions or policies.[29] A small number of recent studies have used DCEs to explore relative preferences for different aspects of financial incentives for healthy behaviours. These find that more flexible payments (e.g. cash) are preferred by potential recipients to those that can only be spent on specific goods (e.g. at a sports shop or venue).[30, 31] However, the range of financial incentive characteristics and health behaviours that have been explored using DCE methods are both limited. Nor has any attempt been made to determine how preferences may vary according to characteristics of respondents. Furthermore, studies have focused specifically on acceptability of financial incentives to potential recipients of financial incentives. In the context of a publically funded healthcare system, such as the UK, where any large scale financial incentive programme is likely to be publically funded, wider acceptability of financial incentives to the general public as a whole, and not just potential recipients, is also important.

We conducted a DCE with the aim to explore relative preferences of UK adults for a range of attributes previously identified as influencing acceptability of financial incentives; as well as whether these preferences varied according to socio-demographic characteristics of respondents. We did not restrict our sample to potential recipients of the financial incentives investigated.

## Methods

Discrete choice experiments describe hypothetical interventions according to their key characteristics, or ‘attributes’ (e.g. type of reward, value of incentive), and ‘levels’ of these attributes (e.g. cash, shopping voucher; higher, lower values). Participants are then asked which of a small number of intervention ‘scenarios’, combining different levels of each attribute, they prefer. This allows relative preferences for attribute levels to be determined. Discrete choice experiments are well-established in health economics[32–34] and increasingly used in public health.[35–37] We followed best practice recommendations for conducting a DCE,[38, 39] collecting data from UK adults in an on-line survey.

### Identification of behaviours, attributes and levels

We focused on four healthy behaviours for which there is evidence that financial incentives can be effective:[10] smoking cessation, regular physical activity, attending a primary care provider for disease screening, and attending a primary care provider for adult vaccination. We used a range of previous research to identify attributes, and levels, of financial incentives that are likely to influence acceptability (see Table 1).[11, 13, 26] In accordance with reporting recommendations for DCEs.[40] the qualitative research used to inform attribute development is reported in full elsewhere.[26] In all cases, attributes and levels were realistic and plausible in policy terms.[38, 39]

**Table 1:** Attributes and levels of financial incentive interventions four health behaviours.

Attribute	Levels for smoking cessation	Levels for regular physical activity	Levels for attending for vaccination	Levels for attending for screening
**Type of incentive**	No reward	No reward	No reward	No reward
	Cash	Cash	Cash	Cash
	Shopping vouchers	Shopping vouchers	Shopping vouchers	Shopping vouchers
	Lottery tickets	Lottery tickets	Lottery tickets	Lottery tickets
**Total value**	£15 over four weeks	£15 over four weeks	£15 for one off attendance	£15 for one off attendance
	£140 over four weeks	£140 over four weeks	£140 for one off attendance	£140 for one off attendance
	£265 over four weeks	£265 over four weeks	£265 for one off attendance	£265 for one off attendance
	£390 over four weeks	£390 over four weeks	£390 for one off attendance	£390 for one off attendance
	£515 over four weeks	£515 over four weeks	£515 for one off attendance	£515 for one off attendance
	£1000 over four weeks	£1000 over four weeks	£1000 for one off attendance	£1000 for one off attendance
**Schedule**	Same value each week	Same value each week	NA	NA
	Value progressively increases	Value progressively increases	NA	NA
**Other information provided**	No other information	No other information	No other information	No other information
	Written leaflet on harms of smoking & ways to quit	Written leaflet on benefits of activity & ways to be more active	Written leaflet on benefits of disease screening	Written leaflet on benefits of vaccination
	Face-to-face discussion on harms of smoking & ways to quit	Face-to-face discussion on benefits of activity & ways to be more active	Face-to-face discussion on benefits of vaccination	Face-to-face discussion on benefits of disease screening
	Written leaflet & face-to-face discussion on harms of smoking & ways to quit	Written leaflet & face-to-face discussion on benefits of activity & ways to be more active	Written leaflet & face-to-face discussion on benefits of vaccination	Written leaflet & face-to-face discussion on benefits of disease screening
**Recipients**	Smokers living in low income households	People living in low income households	People living in low income households	People living in low income households
	Pregnant women smokers	Pregnant women	Pregnant women	Pregnant women
	All smokers	Anyone	Anyone	Anyone

In studies included in systematic reviews,[10, 16] financial incentives commonly take one of three forms: cash, shopping vouchers or lottery tickets. Thus, ‘type of incentive’ was included as an attribute with no reward, cash, shopping vouchers and lottery tickets as levels. Previous evidence suggests that ‘total value’ is a key determinant of acceptability.[30, 31] Levels within the ‘total value’ attribute were set based on the range found in our systematic review of effectiveness of financial incentives,[10] with some smoothing, of £15-£515 (~$US23-$793). We also included one very large incentive value (£1000; ~$US1540) to capture if people could be ‘bought’ into a behaviour at all or if even large amounts would not be effective in motivating a behaviour change.

Contingency Management Theory predicts that gradually increasing the value of incentives, as maintenance of behaviour progresses, leads to more sustained behaviour change.[41] This was captured in a ‘schedule’ attribute. Variable reward schedules can only apply to behaviours that are sustained. Thus, this attribute was not applied to screening and vaccination attendance.

Participants in qualitative studies exploring acceptability of financial incentives often spontaneously identify education, information and support as either alternatives, or complementary, to financial incentives.[26, 35] We therefore included ‘other information provided’ as an attribute with written information, face-to-face discussion or both as levels.

Finally, various potentially vulnerable groups—particularly pregnant women and people living in low income households—have been identified in both qualitative and quantitative work in whom financial incentives may be considered more acceptable.[11, 26, 35] ‘Recipients’ was, therefore, included as an attribute with all eligible people, those living in low income households, and pregnant women as levels.

### Experimental design

**Fig 1:**
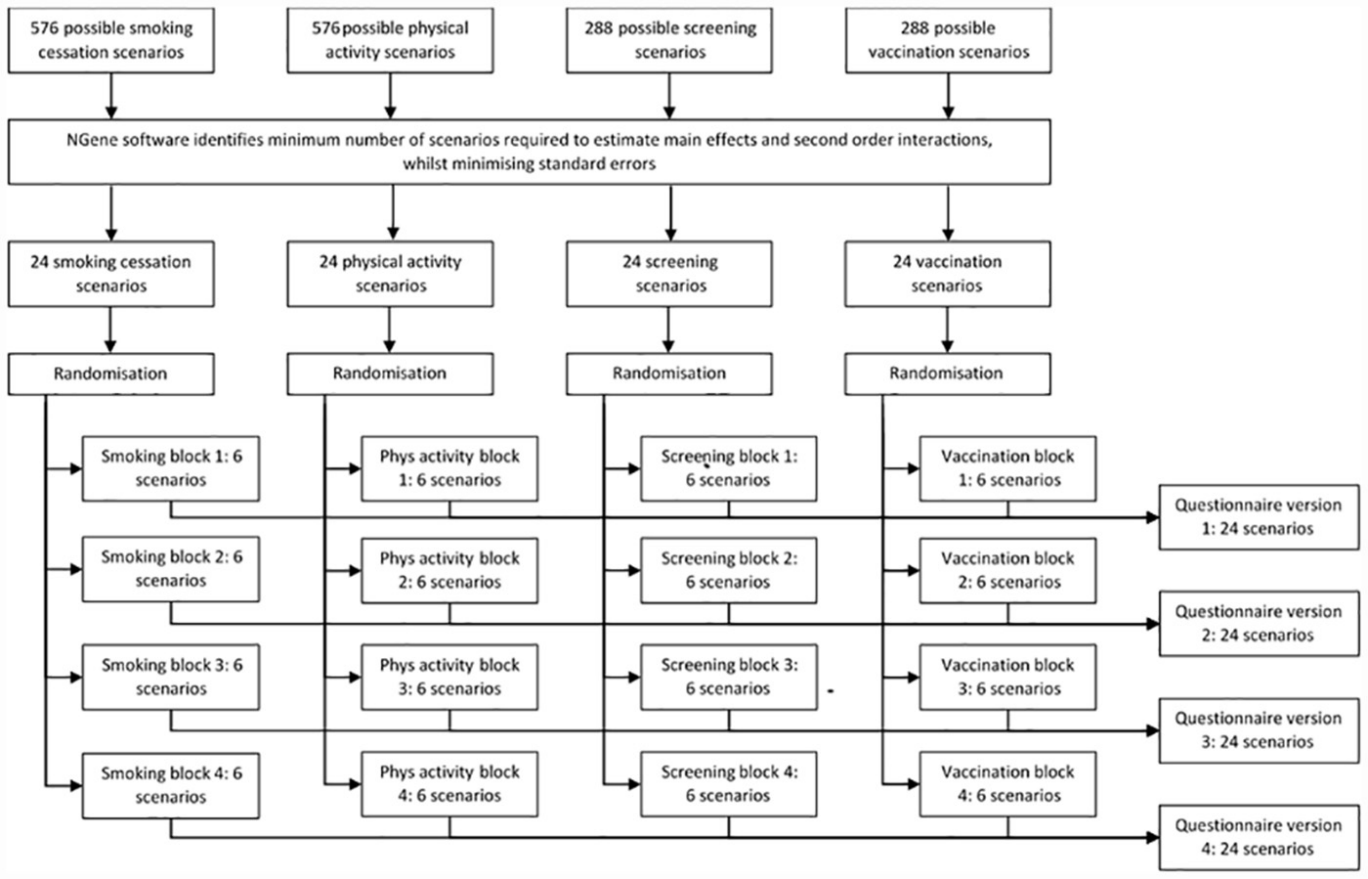
schematic summary of experimental design process.

The experimental design process is summarised in Fig 1. The combination of attributes and levels described in Table 1 would generate 576 unique scenarios (4x6x2x4x3) for smoking cessation and physical activity; and 288 for screening and vaccination (4x6x4x3)– 1728 in total for all four behaviours. This is too many to be considered by any one person. An efficient design was generated using Ngene software[42] to reduce the number of scenarios to the minimum required to estimate main effects and first order interactions, whilst minimising standard errors. This generated 24 pairs of experimental scenarios (‘choice sets’) for each behaviour– 96 across four behaviours and still too many for one person to consider. The 24 choice sets for each behaviour were randomly divided into four blocks of six. One block from each behaviour was then combined to produce four versions of the DCE, each containing 24 choice sets across four behaviours. Participants were randomly assigned to one of these versions.

**Fig 2:**
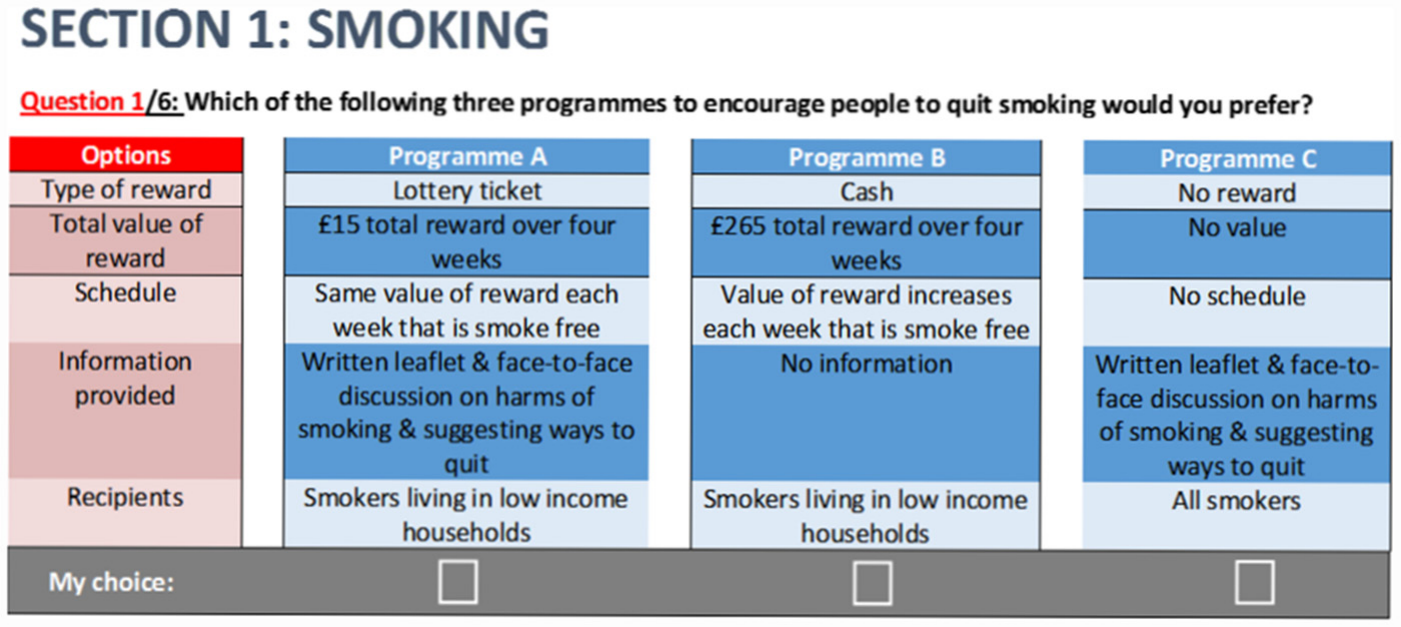
Example choice set.

Each choice set of experimental scenarios was combined with a third scenario including no incentive but both written information and an opportunity for a face-to-face discussion on the benefits of healthy behaviours and strategies for performing them (Fig 2). This represents what might be considered ‘routine’, if not ‘best’, practice for encouraging the healthy behaviours of interest in UK primary care. In all choice sets it was stated that all options were equally effective (to avoid any influence of effectiveness on acceptability) and that programmes would be carefully monitored to avoid ‘gaming’ (i.e. recipients feigning unhealthy behaviour in order to receive rewards for subsequent healthy behaviour).

The full questionnaire included an introduction and instructions, 24 choice sets, and socio-demographic and behavioural questions (age, gender, level of education, current smoking status, and current physical activity level). The 24 choice sets were grouped by behaviour with the order of behaviours randomly allocated across participants.

### Pre-testing and data collection

The draft questionnaire was iteratively pre-tested and refined using cognitive interviewing and the ‘think aloud’ technique.[43, 44] The first author worked through a paper version of the questionnaire with adult volunteers asking them to comment on design, wording and layout and answer all questions, explaining their thought processes as they did so. We conducted three rounds of pre-testing with three participants in each round, making changes to design, wording and layout after each round to maximise respondents’ understanding of the questionnaire.

Main data collection took place via an on-line survey and was conducted by a market research company (ResearchNow) in winter 2014–2015. Participants were invited to take part in the survey via a single-use, personalised, link sent in an email. These prevented participants taking part more than once or sharing links with others.

### Participants and sample size

All participants were aged 18 years or older, normally resident in the UK, and members of ResearchNow’s on-line panel. As per ResearchNow’s normal procedures, participants received small (£2; ~$US3) shopping voucher incentives to take part. Quota sampling was used to maximise the representativeness of participants with quotas set for age, gender, educational attainment, smoking status and physical activity levels (with strata as detailed in Table 2) reflective of the current UK adult population. Respondents who did not complete the full questionnaire were excluded and additional participants recruited to replace them.

**Table 2:** Characteristics of participants, and comparison to UK adult population.

Characteristic	Level	Study sample, n(%); (N = 356)	UK adult population, %^a^
Age	18–29	49 (13.8)	17.6
	30–39	55 (15.5)	16.9
	40–49	77 (21.6)	18.4
	50–59	63 (17.7)	16.8
	60–69	62 (17.4)	14.3
	70–79	38 (10.7)	9.7
	80+	12 (3.4)	10.6
Gender	Male	181 (50.8)	49.2
	Female	175 (49.2)	50.8
Educational attainment	No qualifications	35 (9.8)	23.2
	Secondary school leaving qualifications (e.g. GCSE)	116 (32.6)	29.3
	University entry qualifications (e.g. A-levels, NVQ)	90 (25.3)	12.1
	University degree	114 (32.0)	27.0
Cigarette smoking	Current smoker	46 (12.9)	20.5
	Ex-smoker	72 (20.2)	25.5
	Never smoker	237 (66.6)	54.0
Physical activity	Regularly physically active^b^	85 (23.9)	37.5
	Not regularly physically active	270 (75.8)	62.5

^a^Data on age and gender distribution from 2014 mid-year estimates;[45] data on education attainment from 2011 Census;[46] data on cigarette smoking from Health Survey for England 2013;[47] data on physical activity from Health Survey for England 2012.[48]

^b^Moderately active for 30 minutes or longer on 5 or more days in last week.

We aimed to collect data from 400 participants. Previous studies have highlighted the difficulties of DCE sample size calculations, as sample size calculations are dependent on knowledge of the true choice probabilities—which are not known prior to undertaking research.[38] Health-related DCEs have included samples ranging from 50 [49] to almost 4000.[50] In practice, DCE sample size estimates are generally based on rules-of-thumb, such as a minimum of 10 observations per parameter, plus 50. With five attributes and up to four interaction terms (see below), this would give a minimum required sample size of 140. Thus, we estimated that our target sample of around 400 would be more than sufficient.

### Data analysis

Data was analysed using a random utility model framework and conditional logistic regression to estimate the mean change in utility that respondents placed on attribute levels compared to the reference level (see Box 1). Results are presented as ‘marginal utility values’ for each attribute level, compared to a reference level. Marginal utility values indicate relative preferences for levels within an attribute (for example, relative preferences for cash, shopping vouchers, or lottery tickets compared to no reward). Positive marginal utility values indicate an attribute level is preferred more than the reference level and negative marginal utility values that the attribute level is preferred less than the reference level. Marginal utility values do not imply any quantifiable results other than a ranking of levels compared to a reference level according to the magnitude of the coefficient. P-values are used to identify which differences are statistically significant.

**Box 1. Data analysis**

Data was analysed using a random utility model framework and conditional logistic regression to estimate mean change in utility, value or preference, which respondents placed on an attribute level compared to the reference level. This assumes that the choices individuals make in a DCE reveal the utility they place on the alternatives presented. In a DCE it is assumed that an individual will choose an alternative in a given choice set if the utility derived from that alternative is greater than from any other alternative offered in the choice set.[51]

The utility derived from the alternative chosen is assumed to comprise of two parts: a systematic, observable component; and a stochastic, unobservable component.[52] This can be expressed as:

U = V + ε

Where:

U is the utility derived by an individual,

V is the observable component of this, and

ε is the unobservable component.

In practice, the observable component (V) is captured through the choices respondents make when answering DCE questions. Or:

$$\text{U }=\;\alpha +\;\beta_{1}{\text{X}}_{1}+\;\beta_{2}{\text{X}}_{2}+\;\ldots +\;\beta_{\text{n}}\left(\alpha \text{Z}\right)\;+\;\varepsilon$$

Where:

α is the alternative specific constant (ASC)

X are attributes included in the DCE

β are the parameters (or coefficients) to account for the marginal utility of that attribute

αZ are interaction terms between the ASC and individual characteristics (age, gender, education, smoking status, physical activity)

Interaction terms were used to explore whether preferences for attributes were correlated with each other (no such interactions were found); and whether preferences for attributes varied by respondents’ age, gender, level of education or (in the case of preferences for smoking cessation and physical activity), current behaviour.

### Ethics and data sharing

Ethical approval was obtained from Newcastle University’s Faculty of Medicine’s Research Ethics Committee (reference 00775_1). Participants were provided with written information on the study before deciding to take part and indicated their consent to take part by clicking a button before data collection took place. No personally identifying data were collected. As part of the written information and consent procedure, participants were informed that their data would not be shared until three years after collection. For this reason we cannot share data at this time.

## Results

A total of 356 individuals completed the DCE and were included in the analysis—more than twice as many as indicated by our sample size calculation. Data was missing on educational attainment, cigarette smoking status and physical activity for one person. The sampling quotas were not entirely achieved. Compared to the UK adult population, participants were more likely to be aged 30–79 years, had a higher educational attainment, were less likely to be current or ex-smokers, and less likely to be regularly physically active (Table 2).

Marginal utility values from the DCE are presented in Table 3. A statistically significant positive marginal utility value for cash rewards in relation to vaccination indicates that cash rewards were preferred to no rewards for vaccination. However, there were no statistically significant differences in preferences for shopping voucher rewards compared to no rewards for all behaviours, and for cash rewards compared to no rewards for all behaviours except vaccination. In most cases, these rewards are as acceptable as no reward. In contrast, statistically significant negative marginal utility values for lottery tickets across all behaviours indicate that no reward was preferred to lottery ticket rewards in all cases.

**Table 3:** Marginal utility values of attribute levels for financial incentives for four behaviours (N = 356).

		Marginal utility value^1^							
		Smoking cessation		Regular physical activity		Attendance for vaccination		Attendance for screening	
Attribute	Level	Unadjusted	Adjusted	Unadjusted	Adjusted	Unadjusted	Adjusted	Unadjusted	Adjusted
Type of incentive	No reward	Comparator	Comparator	Comparator	Comparator	Comparator	Comparator	Comparator	Comparator
	Cash	0.12	**1.25**^2^	0.22	**1.84**	0.19	**1.65**	**0.25**^2^	**1.64**
	Shopping vouchers	-0.02	**1.13**	-0.02	**1.66**	-0.06	**1.41**	0.002	**1.41**
	Lottery tickets	**-0.35**	**0.75**	**-0.35**	**1.34**	**-0.53**	**0.89**	**-0.38**	**0.98**
Total value	£UK	**-0.0004**	**-0.0004**	**-0.0003**	**-0.0003**	**-0.0002**	**-0.0002**	-0.0001	-0.0001
Schedule	Same value each week	Comparator	Comparator	Comparator	Comparator	Comparator	Comparator	Comparator	Comparator
	Value progressively increases	0.12	0.14	0.01	0.03	NA^3^	NA	NA	NA
Other info provided	No other information	Comparator	Comparator	Comparator	Comparator	Comparator	Comparator	Comparator	Comparator
	Written leaflet	**-0.35**	**-0.37**	**-0.33**	**-0.36**	0.06	0.22	0.06	**0.32**
	Face-to-face discussion	0.01	-0.03	-0.12	-0.17	0.18	**0.41**	**0.28**	**0.45**
	Written leaflet & face-to-face discussion	0.08	0.10	-0.04	-0.01	**0.38**	**-0.84**	**0.42**	**-0.87**
Recipients	All	Comparator	Comparator	Comparator	Comparator	Comparator	Comparator	Comparator	Comparator
	People living in low income households	**-0.49**	**-0.54**	**-0.41**	**-0.46**	**-0.46**	**-0.42**	**-0.46**	**-0.42**
	Pregnant women	**-0.49**	**-0.49**	**-0.65**	**-0.67**	**-0.82**	**-0.83**	**-0.85**	**-0.87**
Interactions	Option C x age	NA	**0.03**	NA	**0.04**	NA	**0.04**	NA	**0.04**
	Option C x gender (female)	NA	Comparator	NA	Comparator	NA	Comparator	NA	Comparator
	Option C x gender (male)	NA	**-0.30**	NA	**-0.35**	NA	**-0.42**	NA	**-0.59**
	Option C x education (no qualifications)	NA	Comparator	NA	Comparator	NA	Comparator	NA	Comparator
	Option C x education (secondary school)	NA	-0.03	NA	0.06	NA	-0.14	NA	**-0.38**
	Option C x education (university entry)	NA	**-0.29**	NA	-0.09	NA	**-0.46**	NA	**-0.53**
	Option C x education (university degree)	NA	-0.14	NA	0.10	NA	-0.11	NA	-0.13
	Option C x smoking status (never)	NA	Comparator	NA	NA	NA	NA	NA	NA
	Option C x smoking status (current)	NA	-0.18	NA	NA	NA	NA	NA	NA
	Option C x smoking status (ex-smoker)	NA	-0.03	NA	NA	NA	NA	NA	NA
	Option C x physical activity (not active)^4^	NA	NA	NA	Comparator	NA	NA	NA	NA
	Option C x physical activity (active)^5^	NA	NA	NA	**-0.25**	NA	NA	NA	NA
Log-likelihood		-2189	-2123	-2211	-2114	-2102	-1995	-2141	-2020

For all models: number of observations: 6,408; number of choice sets: 2,136; number of respondents: 356.

^1^Marginal utility values indicate relative preferences for levels within an attribute. Positive values indicate an attribute level is preferred more than the comparator and negative values that the comparator is preferred more than the level of interest. P-values identify which differences are statistically significant;

^2^Bold typeface indicates statistically significant at p<0.05;

^3^NA: not applicable;

^4^Not moderately active for 30 minutes or longer on 5 or more days in last week;

^5^Moderately active for 30 minutes or longer on 5 or more days in last week.

Incentives of lower value were weakly preferred to those of higher value, except in the case of screening where there was no difference in preference based on incentive value. Respondents had no preferences in terms of whether incentives for longer term behaviour change were the same amount each week or escalated as behaviour change was sustained.

Respondents preferred that incentives were not accompanied by written information for physical activity and smoking; but that they were accompanied by both written information and face-to-face discussions for vaccination and screening. There was a universal, and strong, preference for incentives offered to all eligible individuals, rather than those targeted at individuals living in low income households or pregnant women.

A small number of participants consistently chose the ‘routine practice’ option over either of the financial incentive scenarios. As shown by the interaction terms in Table 3, these people tended to be older, women, and have attained university entry-level qualifications. Adjusting the analysis to take account of consistently choosing the routine care option changed results in relation to ‘type of incentive’ (with all rewards becoming preferable to no reward), but not in respect of other attributes. This suggests that those who consistently chose ‘routine practice’ had a general dis-preference for financial incentives in general, rather than any particular attribute of financial incentives.

No interactions were found between preferences for different attributes. However, some preferences did vary according to respondents’ socio-demographic characteristics. For all four behaviours, model goodness of fit measured by the log-likelihood ratio statistic improved when controlling for individual characteristics (age, gender, education) and current behaviour where information was available (smoking status, physical activity). Table 4 shows how preferences varied by age, gender and level of education for the attributes where main effects were found in Table 3: type of incentive, information provided, and recipients. Older participants were consistently more likely than younger participants to prefer no reward compared to all types of incentives. They were also more likely than younger participants to prefer incentives accompanied by written information and face-to-face discussions and financial incentives offered to all, rather than targeted at particular groups.

**Table 4:** Interactions between socio-demographics and marginal utility values of attribute levels for financial incentives for four behaviours (N = 356).

		Smoking cessation					Regular physical activity				
Attribute	Level	Age	Male gender^a^	No qualifications^b^	Secondary school^b^	University entry^b^	Age	Gender	No qualifications	Secondary school	University entry
Type of incentive	No reward	Comparator	Comparator	Comparator	Comparator	Comparator	Comparator	Comparator	Comparator	Comparator	Comparator
	Cash	**-**^c^	**+**^d^	NS^e^	**+**	NS	**-**	**+**	**-**	NS	NS
	Shopping vouchers	**-**	NS	NS	NS	NS	**-**	**+**	NS	NS	NS
	Lottery tickets	**-**	NS	NS	NS	NS	**-**	**+**	NS	NS	NS
Other information provided	No other information	Comparator	Comparator	Comparator	Comparator	Comparator	Comparator	Comparator	Comparator	Comparator	Comparator
	Written leaflet	NS	NS	NS	NS	NS	**-**	NS	NS	NS	NS
	Face-to-face discussion	NS	NS	NS	NS	NS	**-**	**+**	NS	NS	NS
	Written & face-to-face	**+**	NS	NS	NS	NS	**+**	NS	NS	NS	NS
Recipients	All	Comparator	Comparator	Comparator	Comparator	Comparator	Comparator	Comparator	Comparator	Comparator	Comparator
	Low income households	**-**	**+**	NS	NS	NS	**-**	**+**	NS	NS	NS
	Pregnant women	**-**	NS	NS	NS	NS	**-**	NS	NS	NS	NS
		Attendance for vaccination					Attendance for screening				
Type of incentive	No reward	Comparator	Comparator	Comparator	Comparator	Comparator	Comparator	Comparator	Comparator	Comparator	Comparator
	Cash	**-**	**+**	NS	**+**	NS	**-**	**+**	NS	**+**	NS
	Shopping vouchers	**-**	**+**	NS	**+**	NS	**-**	**+**	NS	**+**	NS
	Lottery tickets	**-**	**+**	NS	**+**	NS	**-**	**+**	NS	**+**	**+**
Other information provided	No other information	Comparator	Comparator	Comparator	Comparator	Comparator	Comparator	Comparator	Comparator	Comparator	Comparator
	Written leaflet	NS	NS	NS	NS	NS	NS	NS	NS	NS	NS
	Face-to-face discussion	**-**	**+**	NS	NS	NS	**-**	NS	NS	NS	NS
	Written & face-to-face	**+**	**-**	NS	NS	NS	**+**	**-**	NS	NS	NS
Recipients	All	Comparator	Comparator	Comparator	Comparator	Comparator	Comparator	Comparator	Comparator	Comparator	Comparator
	Low income households	**-**	NS	NS	NS	NS	**-**	**+**	NS	NS	NS
	Pregnant women	**-**	NS	NS	NS	NS	**-**	**+**	NS	NS	NS

^a^Versus female gender;

^b^Versus university degree;

^c^Statistically significant negative interaction (p<0.05);

^d^Statistically significant positive interaction (p<0.05);

^e^NS: not statistically significant.

Male participants were more likely than female participants to prefer any financial incentive to no reward for all behaviours except smoking cessation, and to prefer cash incentives to no reward for smoking cessation. Men were also more likely than women to prefer face-to-face information for physical activity and attendance for vaccination; and incentives targeted at those living in low income households for all behaviours except attendance for vaccination. There were few consistent differences in preference according to level of education.

## Discussion

### Summary of findings

We conducted a DCE exploring the relative preferences of UK adults for characteristics of financial incentive interventions for healthy behaviours. Uniquely, we asked all participants to answer questions on financial incentives for four different health behaviours in order to compare how preferences varied between behaviours. Unlike previous work, we also explored socio-demographic determinants of preferences.

In the majority of cases, participants considered cash or voucher incentives equally preferable to no incentive (the exception was a significant preference for cash compared to no incentive for attendance for vaccination). However, there was a consistent preference for no financial incentive compared to a lottery ticket incentive. In general, preferences for financial incentives were inversely related to incentive value. Participants preferred financial incentives available to everyone rather than those targeted only at pregnant women or people living in low-income households. Additional written and face-to-face information alongside financial incentives was preferred for vaccination and screening, but not for smoking and physical activity.

A number of consistent differences in preferences were seen according to age and gender, but not educational level of participants. In general, younger people were more likely than older people to prefer any financial incentive to none, incentives targeted to pregnant women or people living in low-income households, and incentives provided without any additional information. Men were more likely than women to prefer any incentive to none, face-to-face discussions alongside incentives for some behaviours, and incentives targeted at those living in low-income households.

### Strengths & limitations of methods

The use of an on-line market research panel is equivalent to a convenience sample. This may not be representative of the population, limiting the generalisability of results. This constitutes a significant limitation of the work. We chose to use this sample because previous DCEs using more population-representative sampling frames (e.g. from the electoral roll) have resulted in very low response rates—which are leads to limited representativeness.[53] It is also worth noting that even sampling frames such as the UK electoral roll are acknowledged to be biased.[54] By using quota sampling we attempted to ensure that participants reflected the UK adult population in terms of age, gender, educational attainment, smoking status and physical activity. However, our quotas were not always attained. Participants were less likely to be at the extremes of age; were more educated; less likely to currently, or have ever, smoked; and less likely to be regularly physically active than the population as a whole. Despite this, the sample was diverse enough to identify differences in preference according to age and gender.

Personalised, single-use links to the survey sent to participants via email prevented individuals taking part in the survey more than once or sharing links with others.

External validity is a substantial concern of DCEs [55, 56]–it is not clear that respondents’ preferences stated during an on-line survey reflect their true preferences if faced with similar choices in real life. However, in the absence of large scale financial incentive programmes, and observed evidence on acceptability, DCEs can provide valuable preliminary information and improve the understanding of behaviours and triggers to behaviour change. Checks for internal validity showed that the estimated parameters were consistent with findings from the literature. Less than 1% of responses consistently chose only Option A or Option B, indicating that that they did not consider trade-offs between the presented scenarios. Thus, the majority of participants appear to have engaged constructively with the task. However, we were unable to distinguish between personal preferences for what participants would like for themselves, and what they would like for society as a whole.

We determined attributes, and levels, of interest from a range of previous research, including a systematic review [11] and focus group interviews.[26] Furthermore, we ensured that all experimental scenarios were realistic and plausible in the UK context. This reflects best practice in DCEs [38, 39] and increases the relevance of our work to both participants and policymakers.

We excluded a number of potentially important attributes that may influence acceptability of financial incentives. In particular, we asked respondents to consider scenarios that were described as equally effective, and closely monitored to minimise ‘gaming’. One previous DCE found that acceptability of financial incentives increased as stated effectiveness increased.[36] This finding is endorsed by other research.[11, 26] Whilst there is little evidence in practice of ‘gaming’ financial incentive interventions,[57, 58] it is a common concern in qualitative work.[26–28] We excluded both effectiveness and gaming as attributes from the DCE because we felt that both an effective intervention and one where gaming was monitored and minimised were prerequisites of a realistic intervention.

### Interpretation of findings

The finding that, in most cases, there was no difference in preference for cash or voucher incentives versus no incentive suggests that financial incentives may not be as unacceptable as previously reported.[11, 24, 26, 27, 35] Indeed, cash incentives were even preferred to no incentives for screening. This may reflect differences in participant groups. Alternatively, it is possible, although unconfirmed, that social desirability bias operates in some research settings such that people feel it would be ‘improper’ or ‘greedy’ to endorse financial incentives in face to face settings. Social desirability bias may be less likely to operate in more anonymous on-line settings.[35] A perception that it is inappropriate to endorse financial incentives may be particular to the UK context where health care does not involve any financial transactions. There is some indication that financial incentives may be more publically acceptable in settings where paying for health care is normalised.[11]

The strong negative preference for lottery-type incentives is interesting given how common these are in research settings, particularly in the USA, and in Quit & Win contests.[10, 22] Our results suggest that lottery incentives for healthy behaviours are unlikely to be acceptable to the UK public. It is not clear if and how cultural and contextual factors influence acceptability of lottery incentives and whether lottery incentives are more acceptable in the USA than the UK—although the prevalence of these type of incentives in the USA suggests so. There is some evidence that UK respondents feel it is ‘unfair’ to be encouraged to take up a healthy behaviour in return for an incentive that you are not certain of receiving.[26]

Our finding of strong negative preferences for incentives targeted at pregnant women or those living in low income households may offer further insight into the negative preference for lottery incentives. Lottery incentives—where only some people who perform the behaviour receive the reward—may be seen as conceptually similar to those targeted at only some population groups. Again, these results may be specific to the UK context where universal entitlement to health care is well established. Future work could directly compare differences in acceptability of financial incentives according to differences in health-care and other aspects of context and culture. Few, if any, attempts have been made to establish if financial incentives are more effective in those living in less socio-economically affluent circumstances.[10] Further research is required to confirm if the effectiveness of financial incentives varies by socio-economic position and how such a finding could be acceptability operationalised.

In qualitative work, pregnant women and those living in low-income households are perceived as most responsive to, and deserving of, financial incentives.[26, 35] This finding is reflected in recent UK research which has focused on financial incentives for breastfeeding and smoking cessation in pregnancy.[28, 37, 58] However, a preference for targeted incentives was not borne out here. It is possible that in some circumstances research participants answer questions on the acceptability of financial incentives from the perspective of society (‘how would I feel if financial incentives were being offered in the UK?’), whilst in other circumstances they answer from the perspective of themselves (‘how would I feel if I were offered a financial incentive?’). Preferences may vary between these two perspectives. Future work should attempt to de-couple preferences for financial incentives from societal and personal perspectives.

The weak preference for lower value incentives, for smoking, physical activity and vaccination, is superficially counter-intuitive. However, this could reflect a common academic concern that external rewards undermine intrinsic motivation—i.e. incentivised behaviours become less attractive.[59] Although a recent analysis suggests that there is little evidence that ‘crowding out’ of internal motivation does occur in relation to financial incentives for healthy behaviours,[60] this has not yet been widely recognised. Qualitative research also reveals concerns that higher value financial incentives are unaffordable in the current financial climate,[26, 27, 35] and this may explain a preference for lower value incentives. There is very little cost-effectiveness evidence on financial incentives for healthy behaviours. However, one study found incentives for smoking cessation in pregnancy had an incremental cost per quality-adjusted life-year of only £482 (~$US744), suggesting they are highly cost-effective.[61]

We found no differences in preferences in relation to incentive schedule. It has been suggested that escalating incentive values is one way to ensure longer term behaviour change.[41] Participants may have considered this a subtlety—particularly as they were explicitly told that both scenarios in each choice set were equally effective.

We included the attribute of additional information because participants in qualitative studies often emphasise the importance of providing education and information on behaviour change as either an alternative to, or alongside, financial incentives.[26, 27, 35] In the current work we found no specific preference for additional written information or opportunities for face-to-face discussions about behaviours for physical activity and smoking cessation. In contrast, there was a positive preference for face-to-face discussions with written information alongside financial incentives for attendance for both vaccination and screening. Respondents may feel that there is less debate over the benefits of smoking cessation[62] and regular physical activity than attending for screening or vaccination; [63–66] and hence that it is more appropriate to discuss these latter behaviours.

Consistent interactions were found between age and gender and preferences for characteristics of financial incentives. Older people were less likely than younger people to prefer any incentive over none, more likely to prefer additional written information and face-to-face discussions alongside incentives, and more likely to prefer incentives available to all, rather than targeted at particular groups. In a cross-sectional sample, it is difficult to know if these differences reflect cohort effects or true age effects. Men were more likely than women to prefer any financial incentive over none, and incentives targeted particularly at those living in low-income households over universal incentives. These age and gender differences may reflect political ideology, with men and older people in the UK being more likely to support right-wing political parties [67]–which promote individual responsibility and less government interference in everyday lives.

Interestingly, there were no clear or consistent differences in preferences for financial incentives according to level of education. This contrasts with clear educational and socio-economic differences in many health related attitudes and behaviours.[47, 68]

### Implications of findings for policy, practice and research

Our results suggest that financial incentives for healthy behaviours are, in general, no less acceptable to the UK public than no incentives. To maximise acceptability, incentives should be in the form of cash or shopping vouchers, and not lotteries; be of low value; and available to all. Incentives for smoking cessation and physical activity would be more acceptable if not accompanied by additional information, whereas combining incentives with written information and the opportunity for face-to-face discussion would be more acceptable for vaccination and screening.

Future research could explore the reasons for some of the differences in preferences reported here compared to qualitative research findings. It is also important to identify how generalisable our results are beyond the UK, and to distinguish between preferences for incentives from the personal and societal point of view. Further information is also required on the cost-effectiveness of financial incentives.

## Conclusions

Preferences amongst UK adults for programmes promoting smoking cessation, physical activity, vaccination and screening did not vary according to whether or not a financial incentive was offered. Financial incentives offering lottery-type rewards and those only available to some population groups were not considered acceptable. Preferences for additional information provided alongside incentives varied between behaviours. Older participants were less likely to prefer any incentive, more likely to prefer additional written information and face-to-face discussions alongside incentives, and more likely to prefer incentives available to all, rather than targeted at particular groups. Men were more likely to prefer any financial incentive over none, and incentives targeted particularly at those living in low-income households over universal incentives. There were no clear differences in preference according to educational attainment.
